# Economic Burden of Cancer for the First Five Years after Cancer Diagnosis in Patients with Human Immunodeficiency Virus in Korea

**DOI:** 10.15430/JCP.2023.28.2.53

**Published:** 2023-06-30

**Authors:** Yoonyoung Jang, Taehwa Kim, Brian H. S. Kim, Jung Ho Kim, Hye Seong, Youn Jeong Kim, Boyoung Park

**Affiliations:** 1 Department of Preventive Medicine, Hanyang University College of Medicine, Seoul, Korea; 2 Program in Regional Information, Department of Agricultural Economics and Rural Development, Seoul National University, Seoul, Korea; 3 Department of Psychology, Sungkyunkwan University, Seoul, Korea; 4 Program in Agricultural and Forest Meteorology, Research Institute of Agriculture and Life Sciences, Seoul National University, Seoul, Korea; 5 Division of Infectious Diseases, Department of Internal Medicine, Yonsei University College of Medicine, Seoul, Korea; 6 AIDS Research Institute, Yonsei University College of Medicine, Seoul, Korea; 7 Division of Infectious Diseases, Department of Internal Medicine, Korea University College of Medicine, Seoul, Korea; 8 Division of Infectious Disease, Department of Internal Medicine, Incheon St. Mary’s Hospital, College of Medicine, The Catholic University of Korea, Incheon, Korea

**Keywords:** Human immunodeficiency virus, Acquired immunodeficiency syndrome, Neoplasms, Death, Cost of illness

## Abstract

This study aimed to estimate the medical cost of cancer in the first five years of diagnosis and in the final six months before death in people who developed cancer after human immunodeficiency virus (HIV) infection in Korea. The study utilized the Korea National Health Insurance Service-National Health Information Database (NHIS-NHID). Among 16,671 patients diagnosed with HIV infection from 2004 to 2020 in Korea, we identified 757 patients newly diagnosed with cancer after HIV diagnosis. The medical costs for 60 months after diagnosis and the last six months before death were calculated from 2006 to 2020. The mean annual medical cost due to cancer in HIV-infected people with cancer was higher for acquired immunodeficiency syndrome (AIDS)-defining cancers (48,242 USD) than for non-AIDS-defining cancers (24,338 USD), particularly non-Hodgkin’s lymphoma (53,007 USD), for the first year of cancer diagnosis. Approximately 25% of the cost for the first year was disbursed during the first month of cancer diagnosis. From the second year, the mean annual medical cost due to cancer was significantly reduced. The total medical cost was higher for non-AIDS-defining cancers, reflecting their higher incidence rates despite lower mean medical costs. The mean monthly total medical cost per HIV-infected person who died after cancer diagnosis increased closer to the time of death. The estimated burden of medical costs in patients with HIV in the present study may be an important index for defining healthcare policies in HIV patients in whom the cancer-related burden is expected to increase.

## INTRODUCTION

The disease burden due to cancer has rapidly increased worldwide due to early detection, aging population, increased prevalence of risk factors, and advancements in medical technology. In 2020, the estimated total numbers of incidence and death of cancer were 19.3 million and 10 million, respectively; in addition, cancer is reported as the first or second leading cause of death in 112/183 countries and the third or fourth leading cause of death in 23 countries [1]. The total global economic burden of cancer from 2020 to 2050 is estimated as 25.2 trillion USD, which is comparable to a tax of 0.55% on gross domestic product per year worldwide [2]. Although direct comparison of this cost is difficult due to different methods or different years for cost estimation, this value has been considerably increased compared to that in 2010, which was estimated as 290 billion USD, including 154 billion USD of medical cost, 67 billion USD of non-medical cost, and 69 billion USD of income loss in 2020 [3].

Previous studies on cancer burden have mostly focused on the total population. However, some groups of people are more vulnerable to cancer. Understanding the economic burden of cancer in this population would help policymakers to establish targeted health policies for specific groups and effectively decrease cancer-related morbidity. These groups include people with human immunodeficiency virus (HIV) infection who are at a higher risk of cancer, particularly infection-related cancer, due to immunosuppression as a consequence of HIV infection [4]. Expensive antiretroviral therapy against HIV is a global issue for HIV control policy [5] and a barrier to access treatment and adherence [6,7]. Thus, HIV-infected people with poor adherence would be more vulnerable to not only the sequelae of HIV infection but also cancer. Therefore, estimating cancer-related economic burden in this vulnerable population would be helpful in cancer management in HIV-infected individuals.

Despite several studies related to HIV treatment cost [6-8], to the best of our knowledge, there have been no studies worldwide estimating the burden on the healthcare system related to cancer treatment of patients with HIV. Therefore, this study aimed to estimate the medical cost of cancer in the first five years of cancer diagnosis and in the last six months of life in patients with HIV infection in Korea, thereby providing key indices of the economic burden associated with cancer in patients with HIV who are highly vulnerable to cancer.

## MATERIALS AND METHODS

This study utilized the Korea National Health Insurance Service-National Health Information Database (NHIS-NHID), which is a mandatory and universal claims database containing information of approximately 97% of the Korean population. The NHIS-NHID comprised information of socioeconomic characteristics, healthcare service usage, health screening, medical expenses for all healthcare services and prescription drugs reimbursed by the NHIS, and death.

First, we selected patients who were newly diagnosed with HIV infection from 2004 to 2020 based on the International Classification of Disease-10th revision (ICD-10) codes (B20-B24) and cost-sharing system code (V103) for HIV infection. The cost-sharing system in Korea is associated with a reduction in out-of-pocket medical expenses for diseases with high financial burden, such as HIV infection, cancer, and rare diseases; thus, the cost-sharing system code is valid for defining diseases. To consider newly diagnosed cases of HIV infection, we excluded patients who received HIV-related medical services between 2002 and2003. To calculate cancer-related medical cost per year after diagnosis, we selected HIV-infected people who were newly diagnosed with cancer. To improve the accuracy of cancer diagnosis, cost-sharing system codes (V193, V194, or V027) for cancer, in combination with the primary diagnosis of cancer based on the ICD-10 codes (C00-C99), were applied. We excluded patients who were diagnosed with cancer before HIV diagnosis by excluding those who received medical services for cancer before HIV diagnosis.

Among 16,671 patients newly infected with HIV from 2004 to 2020, we identified 757 patients who were newly diagnosed with cancer after HIV diagnosis. The newly diagnosed cancer cases included AIDS-defining and non-AIDS-defining cancers. AIDS-defining cancers included Kaposi’s sarcoma (C46), non-Hodgkin’s lymphoma (C82-C86, C96), and cervical cancer (C53) [9]. The remaining cancer types were considered as non-AIDS-defining cancers.

### Medical costs

In this study, only direct medical cost was considered as “cost,” and indirect cost and cost of non-benefited healthcare services, which were not covered by the NHIS, were not considered. To distinguish cancer-related costs from those of HIV or other diseases, all NHIS claims for inpatient, outpatient, and prescription with a cost-sharing system code for cancer (V193, V194, or V027) were defined as cancer-related costs. In the NHIS-NHID, expenses consist of patients’ co-payments and insurers’ payments. The total medical cost for cancer was calculated as the sum of the patients’ co-payments and insurers’ payments.

The total 5-year cancer-related cost from 2006 to 2020 was calculated from the date of cancer diagnosis, and the cost was adjusted for annual variation with a discount rate of 3% each year to standardize all expenses to the currency value in 2020. Subsequently, the monthly average medical cost of cancer from the first month to 60th month was calculated. The individual average total cancer-related medical cost for each year was calculated using the following equation:

$$E_{t}=\sum_{j=1}^{12}P_{tj}C_{tj}$$
*Notation*,*E_t_* = individual average medical cost related to cancer during each year *t* after cancer diagnosis*P_tj_* = survival rate at month *j* during each year *t**C_tj_* = personal average expense of cancer patient at month *j* during each year *t**t* = each year after cancer diagnosis (*t* = 1 to, 5)*j* = month of each year (*j =* 1 to 12)

The individual average monthly medical cost of cancer for 60 months was calculated using the survival rate and monthly expenses from 2006 to 2020, which were adjusted to the discount rate. Monthly survival rates were estimated using non-parametric Kaplan–Meier estimates for censored data, and then monthly survival rates were multiplied by monthly expenses for cancer treatment. The Kaplan–Meier method does not need to divide the period into specific intervals, based on non-parametric statistics, without assuming the distribution of the study population [10]. Considering that a significant part of cancer-related medical costs is incurred within one year of cancer diagnosis, individual average medical cost during the first year of cancer diagnosis was presented as monthly expenses from the first to 12th month of cancer diagnosis.

All cancer (patients) included those who were diagnosed with AIDS-defining or non-AIDS-defining cancers. The identified cancer types were further categorized based on the number of cases for each type, including Kaposi’s sarcoma, non-Hodgkin’s lymphoma, malignant neoplasm of the colorectum, malignant neoplasm of the liver, bile duct, and pancreas, and malignant neoplasm of the stomach. Despite relatively large number of malignant neoplasms of the lung and trachea, their cost was not presented separately because no patient could survive for up to 60 months. Other types of cancer were not sub-categorized because of small number of cases.

To estimate cancer-related medical costs as a total social burden and its trend, the total cancer-related medical cost during the first year of cancer diagnosis by each month was calculated by the summation of the costs associated with all individuals, stratified by the following year ranges: 2006 to 2008, 2009 to 2011, 2012 to 2014, 2015 to 2017, and 2018 to 2020. In addition, for patients who developed cancer after HIV diagnosis and died, individual average total medical cost (not restricted to cancer-related medical costs) for six months before death was presented. The estimated medical costs in period t were converted into US dollars according to the average 2020 exchange rate (1 USD = 1,180 KRW). The study was approved by the Institutional Review Board of Hanyang University, Korea (approval no: HYUIRB-202111-005). We received permission to analyze NHIS-NHID using pseudonymized information. This study complied with the regulations of the Reporting of Observational Studies in Epidemiology for cohort studies.

## RESULTS

**Figure 1:**
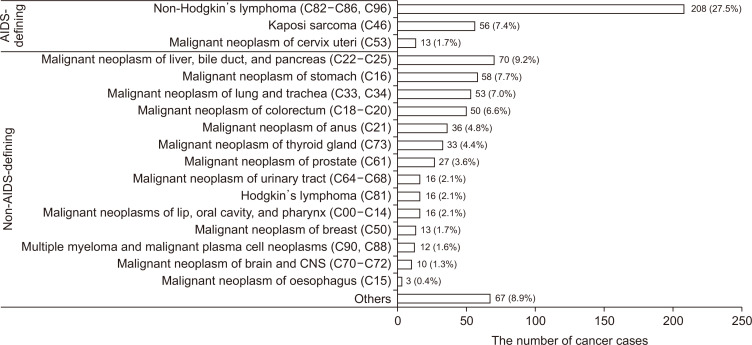
Type of newly incident cancer in patients with human immunodeficiency virus (HIV) in Korea. AIDS, acquired immune deficiency syndrome.

Of the 757 patients newly diagnosed with cancer after HIV diagnosis, AIDS-defining cancer, including 208 non-Hodgkin’s lymphoma, 56 Kaposi sarcoma, and 13 cervical cancer, accounted for 36.6% (n = 277; Fig. 1). Non-AIDS-defining cancers, which accounted for 63.4% of cancer developed in HIV patients included 70 cases of hepatobiliary and pancreatic cancer (9.2%), 58 stomach cancer (7.7%), 53 lung and tracheal cancer (7.0%), and 50 colorectal cancer (6.6%).

**Figure 2:**
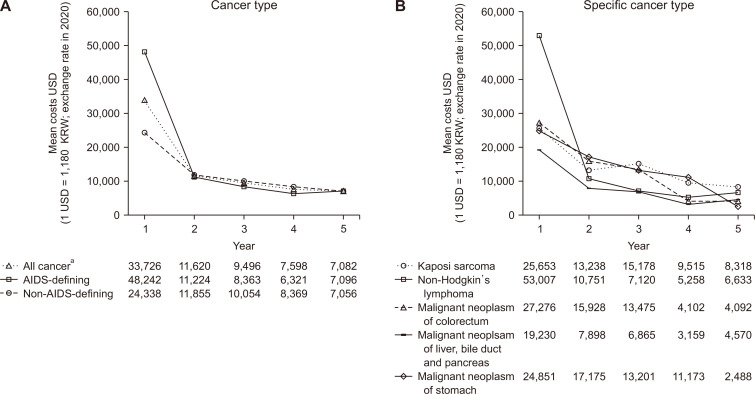
Mean annual medical cost due to cancer per patient in the first five years following cancer diagnosis in patients with human immunodeficiency virus. ^a^All cancer (patients) included those who were diagnosed with acquired immune deficiency syndrome (AIDS)-defining or non-AIDS-defining cancers.

The mean annual medical cost due to cancer per patient in the first five years following cancer diagnosis for all cancers, AIDS-defining cancers, and non-AIDS-defining cancers, are presented in Figure 2. In all three groups, medical costs were the highest in the first year of cancer diagnosis and decreased significantly in the second year. Compared with cancer costs in the first year, the mean medical cost due to cancer in the second year decreased by 65.5% (33,725 USD to 11,620 USD) for all cancers, 76.7% (48,242 USD to 11,224 USD) for AIDS-defining cancers, and 51.3% (24,338 USD to 11,855 USD) for non-AIDS-defining cancers. Although the cost decreased over the years following cancer diagnosis, the decrease after the second year was less significant. In the first year, the mean annual medical cost was approximately two-fold higher for AIDS-defining cancers than that for non-AIDS-defining cancers; however, there was no significant difference in the mean annual medical cost from the second to fifth year between the two groups (Fig. 2A). Similarly, the mean annual medical cost per patient stratified by the cancer type was the highest in the first year and largely decreased in the second year. In the first year, non-Hodgkin’s lymphoma showed a significantly higher mean annual medical cost (53,007 USD) than that of other cancer types (less than 30,000 USD). In the second year, the mean annual medical cost of non-Hodgkin’s lymphoma (10,751 USD) decreased by 79.7% compared to that in the first year and was lower than that of other types of cancers (Fig. 2B).

**Figure 3:**
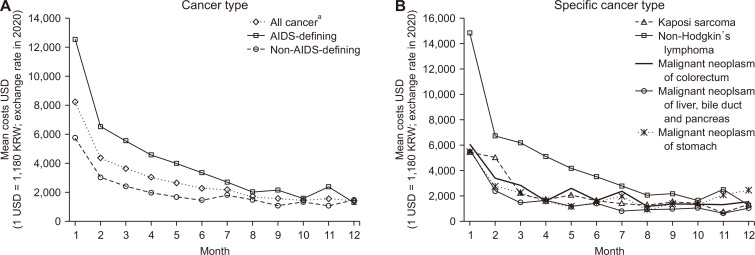
Mean monthly medical cost due to cancer per patient in the first 12 months following cancer diagnosis in patients with human immunodeficiency virus. ^a^All cancer (patients) included those who were diagnosed with acquired immune deficiency syndrome (AIDS)-defining or non-AIDS-defining cancers.

Figure 3 shows the mean monthly medical costs of cancer per patient during the first 12 months of cancer diagnosis. The AIDS-defining cancer group showed a mean monthly medical cost of 12,461 USD in the first month, which was 2.2-times higher than that of the non-AIDS-defining cancer group (5,679 USD). The mean monthly medical cost for AIDS-defining cancers and non-AIDS-defining cancers in the second month decreased by 47%-48% compared to that in the first month. When analyzed by the cancer type, all cancer types showed the highest mean medical cost for the first month after diagnosis, followed by a significant decrease in the second month. The mean monthly medical cost of non-Hodgkin’s lymphoma was the highest in almost each of the first 12 months among all cancer types, which was 14,833 USD and 6,729 USD in the first and second months after diagnosis, respectively (Appendix 1).

**Figure 4:**
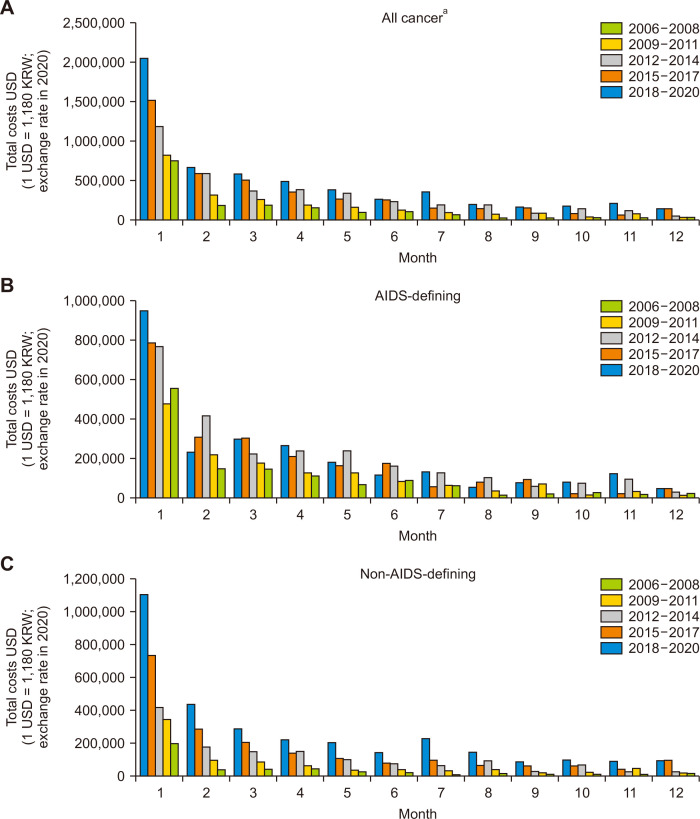
Total monthly medical cost due to cancer in the first 12 months following cancer diagnosis in patients with human immunodeficiency virus. ^a^All cancer (patients) included those who were diagnosed with acquired immune deficiency syndrome (AIDS)-defining or non-AIDS-defining cancers.

The total medical cost due to cancer for the first year of cancer diagnosis in patients with HIV infection was 17,799,522 USD in 2006 to 2020. The total medical costs due to cancer per month for the first year of cancer diagnosis in patients with HIV infection in the year 2006 to 2008, 2009 to 2011, 2012 to 2014, 2015 to 2017, and 2018 to 2020 are shown in Figure 4. For the non-AIDS-defining cancer group, the total monthly medical cost during the first 12 months was higher in recent years (2018 to 2020) and decreased in the remote year groups, despite standardization using the discount rate. However, for the AIDS-defining cancer group, there was no consistent pattern in the total monthly medical cost according to the year of diagnosis, except in the first month, in which the highest cost was shown in recent years (Appendix 2).

**Figure 5:**
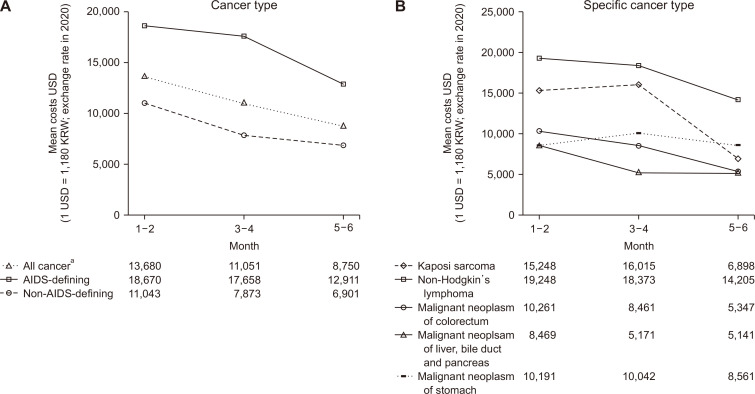
Mean medical cost including the cost of cancer treatment and other treatments per patient in the last six months of life in patients with human immunodeficiency virus and diagnosed with cancer. ^a^All cancer (patients) included those who were diagnosed with acquired immune deficiency syndrome (AIDS)-defining or non-AIDS-defining cancers.

Figure 5 shows the mean medical cost (for every two months) for all healthcare services, including the cost of cancer treatment and other treatments for each cancer patients with HIV infection in the final six months before death. The mean medical costs increased closer to the time of death in the AIDS-defining, non-AIDS-defining, and specific cancer groups. Compared to the non-AIDS-defining cancer group, the AIDS-defining cancer group showed a 69.1% (18,670 USD vs. 11,043 USD), 124.3% (17,658 USD vs. 7,873 USD), and 87.1% (12,911 USD vs. 6,901 USD) increase in mean medical costs in the last 1 to 2 months, 3 to 4 months, and 5 to 6 months before death, respectively. Comparison among groups according to the cancer type showed a higher medical cost burden in all final months of life in the non-Hodgkin’s lymphoma group than that in the other cancer groups.

## DISCUSSION

In the present study, based on nationwide insurance claim data, we estimated the annual direct medical cost due to cancer in the first five years following cancer diagnosis, monthly medical cost due to cancer in the first 12 months of cancer diagnosis, and total medical cost in the last six months of life in all cancers, AIDS-defining cancers, non-AIDS-defining cancers, and specific cancer groups in patients with HIV infection, one of the most vulnerable populations to cancer. To the best of our knowledge, this study is the first to estimate cancer-related medical costs as an important chronic disease in HIV-infected people at the personal and social levels.

The main findings of this study can be summarized as follows: first, we identified that the mean annual medical cost due to cancer in HIV-infected people was higher in AIDS-defining cancers (48,242 USD) than that in non-AIDS-defining cancers (24,338 USD), particularly in non-Hodgkin’s lymphoma (53,007 USD) for the first year of cancer diagnosis; from the second year, the mean annual medical cost due to cancer was significantly reduced and differences in the mean annual medical cost of cancer were not observed according to the cancer type. Second, the highest medical cost for the first year of cancer diagnosis was incurred during the first month of cancer diagnosis. Third, total medical cost, which was affected by both mean medical cost and number of cancer cases, was higher for non-AIDS-defining cancers, reflecting higher incidence rate of non-AIDS-defining cancers, considering their lower mean medical costs. Fourth, the mean monthly total medical cost per HIV-infected person who died after cancer diagnosis increased closer to the time of death.

Although a direct comparison would be difficult due to the different time periods and definitions of medical costs between studies, we observed that the average annual medical cost per cancer patient in HIV-infected individuals tended to be higher than that in the general population [11,12]. In the general population, the average medical expenditure for patients with non-Hodgkin’s lymphoma per person during the first to fifth year was 16,241,853 KRW, 7,266,744 KRW, 5,711,032 KRW, 4,430,109 KRW, and 3,450,786 KRW, respectively, which is one-fourth to one-fifth that of patients with non-Hodgkin's lymphoma and concurrent HIV infection. In addition, the medical cost of colorectal or liver cancer in HIV-infected people was two to three times higher than that of the general population with the same cancer site in Korea [12].

Studies have suggested three clinically relevant stages of cancer care: initial phase, defined by the first 6 or 12 months after diagnosis; terminal phase, defined by the final 12 months before death; and continuing phase, defined as the period between initial phase and terminal phase. Thus, the cost of cancer care continuum according to the phases of cancer case has been estimated [13-15]. Regarding end-of-life care for cancer, studies have considered several time cut-offs, such as 3, 6, or 12 months before death [13-20]. The initial and terminal phases had a higher burden of medical costs than that of the continuing phase, showing an overall U-shaped pattern [13,15]. Our findings showed a comparable pattern of expenses in medical costs due to cancer for AIDS-defining cancer, non-AIDS-defining cancer, and each cancer type in patients who developed cancer after HIV diagnosis.

None of the previous studies estimated cancer cost by month; our study revealed that the mean monthly cost of cancer per patient was the highest in the first month of cancer diagnosis and then decreased by approximately 45% to 60% in the second month. The percentage of medical costs due to cancer in the first month of the first year of cancer diagnosis was 24.2% for all cancers, 25.8% for AIDS-defining cancers, and 23.3% for non-AIDS-defining cancers. The results suggest that medical support for cancer in HIV-infected people should be focused during the earlier period of cancer diagnosis.

High medical cost of cancer during the initial phase in recent years may be attributable to high cost associated with anticancer therapy [21-23]. In another aspect, recent increase in the total medical cost of cancer in terms of social burden can be attributed not only to the increased cost but also to the increasing number of cancer patients infected with HIV. A previous study also suggested an increased cancer burden in patients with HIV infection, and that the burden of non-AIDS-defining cancer would be increased and that of AIDS-defining cancer would be decreased due to aging and improved therapy for HIV infection [24]. In this study, the number of HIV patients with cancer was 235 in 2018 to 2020, 177 in 2015 to 2017, 152 in 2012 to 2014, 108 in 2009 to 2011, and 85 in 2006 to 2008. The increased total medical cost due to non-AIDS-defining cancers in recent years would reflect this.

Previous studies on the general population reported increased medical costs in the final months of the life of patients [16,18-20]. In Korea, cost-sharing systems to reduce out-of-pocket medical expenses for cancer are available for five years after cancer diagnosis; hence, there are difficulties in estimating the medical cost of cancer after five years of diagnosis. Thus, although we evaluated the burden of medical costs in the last six months of life, we could not distinguish the cost of cancer treatment from the total medical cost; thus, the cost included all health care services, in addition to cancer treatment. Our results showed that the mean total medical cost per patient in the last two months of life was similar to that in the initial phase. When the total medical cost per patient in the last six months of life was assessed in 2-month increments, the total medical cost was the highest in the last two months of life and tended to decrease in months that were farther from the time of death.

There are several limitations of our study that must be considered while interpreting its findings. First, cost considered in this study did not include the cost of non-benefited healthcare services and prescription drugs that were not covered by the NHIS. Despite expanding health insurance coverage for cancer treatment, approximately 29.0% of healthcare services in cancer patients in 2006 were not covered by the NHIS, and 19.8% of these services in 2021 are still not covered by the NHIS [25]. Thus, medical cost estimated using the NHIS may underestimate the actual medical cost of each patient. In addition, considering the coverage rate of cancer treatment in previous years was lower than that in recent years, medical cost of cancer in previous years may be underestimated to a greater extent than that in recent years. Second, HIV-infected people are generally diagnosed with cancer at an advanced stage due to a lower uptake of screening or limited medical access for primary or secondary prevention [9,26] than the general population.

Patients with advanced cancer at diagnosis have higher medical costs than those with early-stage cancer [27-29]. Because the NHIS-NHID data did not include information on cancer stage, we could not consider stage-specific cancer costs and their comparison with estimated cost in the general population. Third, previous studies have defined the initial phase as the period spanning 6 to 12 months after cancer diagnosis and reported that the cancer-related medical expenses are high in the initial phase and then decreases over time [13-15]. In contrast to previous studies, our research focused on analyzing the monthly average medical expenses per patient spent on a monthly basis from the time of cancer diagnosis. Our findings revealed that approximately 25% of the average medical expenses per patient within the first year after cancer diagnosis were spent in the first month. However, we were unable to determine the details of the medical expenses. Further investigations are warranted to elucidate the types of medical services that are associated with high medical expenses in the first month after cancer diagnosis. Fourth, this study considered medical costs of cancer from the NHIS for a long period (2006-2020).

The quality of information reported to the NHIS varies over time according to changes in insurance policies. For example, the proportion of cancer cost-sharing by the NHIS decreased from 30% to 10% in 2005 and from 10% to 5% in 2009 [30]. Thus, we showed the cost of adding patients’ co-payments and insurers’ payments to avoid the impact of changes in the cost-sharing system. However, other changes, such as reporting policies, are not reflected. Fifth, several studies have provided separate estimations of the direct and indirect medical costs of cancer in assessing the burden of cancer and reported high economic burden of indirect medical costs, including production loss, cost of care, and transportation expenses [31-33].

In this study, we focused on changes in medical costs from the date of cancer diagnosis and did not consider indirect medical costs of cancer. Further studies considering both direct and indirect economic burden of cancer in HIV-infected people, including premature death followed by productivity loss, are warranted. However, the estimated medical cost of cancer in this study may be a simple and adequate index to understand the economic burden of cancer in patients with HIV who are at a high risk for cancer.

The burden of medical costs in patients with HIV estimated in the present study may be an important index for establishing healthcare policies for HIV patients in whom cancer-related burden is expected to increase [24]. Further studies to estimate future cancer burden in people infected with HIV, including projections of cancer incidence, mortality, and cancer-related costs, including both direct and indirect costs, are warranted.
