# Supplementary material for: Economic Burden of Cancer for the First Five Years after Cancer Diagnosis in Patients with Human Immunodeficiency Virus in Korea
Source: J Cancer Prev. 2023 Jun 30;28(2):53–63. doi: 10.15430/JCP.2023.28.2.53 (PMC10331032; doi:10.15430/JCP.2023.28.2.53)
Supplement: Supplementary file 1 [file jcp-28-2-53-appendix.pdf]

Appendix 1. The mean monthly medical cost of cancer per patient in the first 12 months of cancer diagnosis in patients with human immunodeficiency virus

Unit: USD (1 USD = 1,180 KRW; exchange rate in 2020)

Month	Cancer type			Specific cancer type				
	All cancer[a]	AIDS-defining	Non-AIDS-defining	Kaposi sarcoma	Non-Hodgkin's lymphoma	Malignant neoplasm of colorectum	Malignant neoplasm of liver, bile duct, and pancreas	Malignant neoplasm of stomach
1	8,158	12,461	5,679	5,451	14,833	6,084	5,674	5,494
2	4,314	6,490	3,017	5,041	6,729	3,399	2,420	2,767
3	3,623	5,492	2,399	2,141	6,191	2,832	1,457	2,263
4	2,996	4,534	1,953	1,688	5,115	1,601	1,654	1,541
5	2,623	3,949	1,677	2,080	4,177	2,599	1,184	1,136
6	2,265	3,320	1,436	1,616	3,516	1,638	1,420	1,503
7	2,149	2,677	1,780	1,393	2,789	2,370	810	2,002
8	1,668	1,998	1,473	1,276	2,055	1,175	920	869
9	1,550	2,136	1,076	1,522	2,183	1,371	963	1,418
10	1,429	1,554	1,325	1,429	1,637	1,347	1,053	1,315
11	1,560	2,358	1,062	678	2,519	1,314	636	2,093
12	1,390	1,273	1,463	1,340	1,263	1,545	1,038	2,451

[a]All cancer (patients) includes those who were diagnosed with acquired immune deficiency syndrome (AIDS)-defining or non-AIDS-defining cancer.

Appendix 2. The total monthly medical cost of cancer in the first 12 months following cancer diagnosis in patients with human immunodeficiency virus

Unit: USD (1 USD = 1,180 KRW; exchange rate in 2020)

Type	Month	2006-2008	2009-2011	2012-2014	2015-2017	2018-2020
All cancer[a]	1	755,074	823,837	1,187,021	1,522,329	2,055,725
	2	184,465	315,477	591,629	592,179	665,491
	3	185,060	263,111	371,206	506,642	586,476
	4	154,045	187,718	388,554	350,811	486,380
	5	93,143	163,448	337,781	268,303	385,277
	6	107,097	121,460	235,705	252,407	259,301
	7	68,463	96,234	191,409	151,644	358,955
	8	29,371	75,056	193,734	144,030	197,849
	9	29,374	89,681	87,050	154,382	162,834
	10	34,987	36,957	142,221	82,390	176,154
	11	27,743	78,862	118,348	63,757	211,513
	12	34,743	30,409	51,810	143,280	139,131
AIDS-defining	1	557,761	478,012	769,098	786,872	949,613
	2	147,242	220,672	415,426	305,999	230,758
	3	144,547	177,296	223,084	302,469	298,147
	4	110,527	126,963	239,314	212,287	266,402
	5	67,019	127,764	238,518	163,359	180,888
	6	87,349	83,305	161,573	175,246	116,258
	7	61,112	62,879	126,496	55,744	131,605
	8	14,061	35,938	101,812	79,803	53,876
	9	20,247	69,603	58,049	93,110	76,706
	10	25,587	15,169	74,477	21,845	79,322
	11	18,006	32,938	94,382	22,014	122,402
	12	21,220	13,501	27,912	46,761	46,945
Non-AIDS-defining	1	197,313	345,825	417,922	735,457	1,106,112
	2	37,223	94,805	176,203	286,181	434,733
	3	40,513	85,815	148,122	204,173	288,330
	4	43,518	60,756	149,240	138,524	219,978
	5	26,123	35,684	99,262	104,944	204,389
	6	19,748	38,155	74,133	77,161	143,043
	7	7,351	33,356	64,913	95,900	227,350
	8	15,310	39,118	91,922	64,227	143,973
	9	9,127	20,078	29,001	61,272	86,128
	10	9,400	21,788	67,743	60,545	96,832
	11	9,737	45,924	23,966	41,742	89,111
	12	13,523	16,908	23,898	96,518	92,185

[a]All cancer (patients) includes those who were diagnosed with acquired immune deficiency syndrome (AIDS)-defining or non-AIDS-defining cancer.
